# Nestin and SOX2 Maintain self-renewal Abilities of Different Pancreatic Cancer Stem Cell Populations

**DOI:** 10.1007/s12015-025-11006-3

**Published:** 2025-10-23

**Authors:** Lisa-Marie Philipp, Patrick Hoffmann, Luisa Hattingen, Amelie Modi, Susanne Sebens

**Affiliations:** Institute for Experimental Cancer Research, Kiel University and University Hospital Schleswig-Holstein Campus Kiel, Kiel, Germany

**Keywords:** Pancreatic ductal adenocarcinoma, Cancer stem cells, Plasticity, Epithelial-to-Mesenchymal transition, Chemoresistance, Invasion, Metastasis

## Abstract

Metastasis and therapy resistance are main clinical challenges of pancreatic ductal adenocarcinoma (PDAC) still limiting patient`s prognosis. Both are dependent on tumor cell plasticity, which allows rapid adaptation of tumor cells to changing microenvironmental conditions. Epithelial-Mesenchymal-Transition (EMT), a process by which carcinoma cells acquire invasive abilities, is associated with a gain of cancer stem cell (CSC) properties. Different CSC phenotypes were described in PDAC, whereby high levels of the CSC marker Nestin was identified in CSC clones of mesenchymal Panc1 cells, while CSC clones of epithelial Panc89 cells were characterized by a high SOX2 expression. To investigate the functional impact of these CSC markers in PDAC cells with different EMT phenotypes, expression of either CSC marker was silenced in heterogenous (parental) and CSC PDAC populations to analyze their impact on essential malignancy associated properties.

SiRNA-mediated knockdown (KD) of *NES* and/or *SOX2* in Panc1 and Panc89 cell variants (parental and CSC population), respectively, was successfully achieved. Decreased *NES* expression in Panc1 cell variants and decreased *SOX2* expression in Panc89 cell variants significantly inhibited self-renewing properties, however, only marginally impacted cell growth, EMT marker expression, migration and invasion properties as well as response to chemotherapy. Overall, our data indicate that Nestin and SOX2 are crucial mediators of self-renewal capabilities of mesenchymal and epithelial PDAC cell variants, respectively, but that further factors are required for the maintenance of other malignancy associated properties.

**Graphical Abstract:**

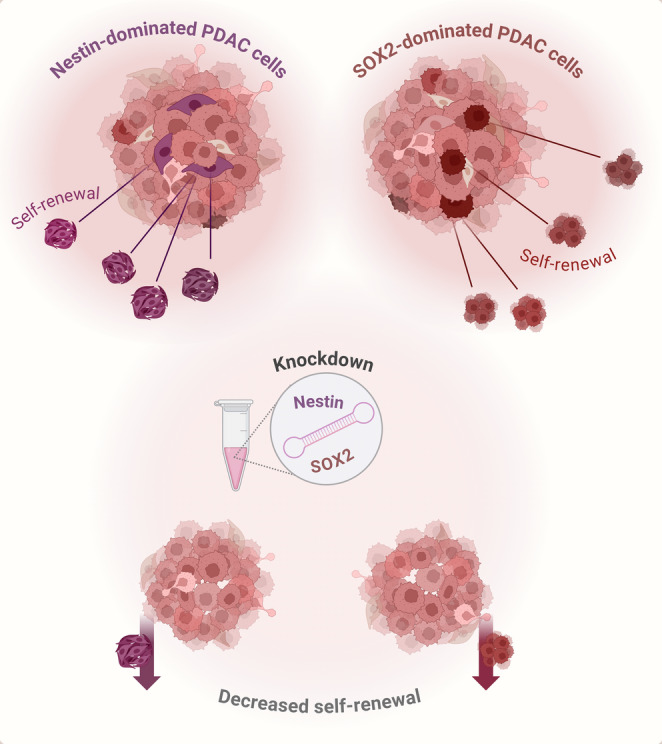

**Supplementary Information:**

The online version contains supplementary material available at 10.1007/s12015-025-11006-3.

**Abbreviations**: BSA: Bovine serum albumin; CFA: Colony-formation assay; CSC: Cancer stem cell; CO_2_: Carbon dioxide; CTRL: Control; CTRLsi: Control siRNA; EDTA: Ethylenediaminetetraacetic acid; EMT: Epithelial-to-Mesenchymal Transition; FCS: Fetal calf serum; GAPDH: Glycerinaldehyde-3-phosphate-Dehydrogenase; HCL: Hydrochloric acid; Holo: Holoclone cells; IFS: Immunofluorescence staining; KD: Knockdown; MET: Mesenchymal-to-Epithelial Transition; ON: Overnight; Para: Paraclone cells; PBS: Phosphate buffered saline; PCR: Polymerase chain-reaction; PDAC: Pancreatic ductal adenocarcinoma; PFA: Paraformaldehyde; RNA: Ribonucleic acid; RPMI: Roswell Park Memorial Institute; RT: Room temperature; RT-qPCR: Quantitative real-time polymerase chain-reaction; SC: Stem cell; SCC: Single-cell cloning; SD: Standard deviation; SEM: Standard error of means; siRNA: small-interfering ribonucleic acid.

*Graphical abstract*.

## Introduction

Pancreatic ductal adenocarcinoma (PDAC) is one of the most lethal cancer entities [1, 2]. The high mortality of PDAC is represented by a poor 5-year survival rate of only about 11%, which is often due to the lack of early and specific symptoms as well as early distant metastasis to liver, lung and peritoneum [1, 3–6]. The only curative treatment option is the R0 resection of the primary tumor together with e.g. adjuvant or neoadjuvant chemotherapy [7]. However, only about 20% of the patients are eligible to undergo surgery because of an already advanced or metastasized disease stage at time point of diagnosis [7–12].

The multistep process of metastasis is initiated by the dissemination of tumor cells from the primary tumor, followed by entering the circulation and finally, proliferation of the tumor cells and outgrowth to clinically overt metastases at secondary sites. The latter only occurs if cancer cells exhibit a high cellular plasticity as they have to repeatedly switch their phenotype, e.g. from a proliferative to an invasive stage and back [13–15]. The Epithelial-to-Mesenchymal Transition (EMT) is one process by which carcinoma cells can acquire motile and invasive traits [16, 17]. EMT is characterized by the loss of differentiation due to downregulated adhesion molecules and deprivation of epithelial proteins, like E-cadherin and an increase of mesenchymal markers, like L1CAM and Vimentin [18–23]. In this context, the transcription factors ZEB1, ZEB2 and OVOL2 are important regulators of cellular plasticity [16, 17, 24–29]. Additionally, the process of EMT is associated with the gain of cancer stem cell (CSC) properties [30–35]. By their self-renewal capacity, resistance to chemotherapy, as well as their ability to give rise to different cancer cell clones, CSCs play a key role in PDAC progression and metastasis [32, 36–38]. Adding to the cellular plasticity, CSC properties can be gained and lost in response to the environmental conditions [33–42]. CSC plasticity might also contribute to tumor cell heterogeneity of PDAC thereby potentially limiting therapy responses of PDAC patients [43, 44]. Underscoring the heterogeneity of CSC populations, several markers have been used for the identification of CSCs in PDAC, e.g. Nestin and SOX2 [43, 45–47]. The intermediate filament Nestin and the stem cell (SC)-transcription factor SOX2 have been shown to play a role in the maintenance of CSC features in different cancer entities [48, 49]. Nestin affects cell motility and EMT properties in PDAC cells and its knockdown (KD) has led to reduced tumor incidence and volumes as well as decreased liver metastasis in a murine PDAC model [50, 51]. Since elevated SOX2 expression is rarely detected in pancreatic intraepithelial neoplasia, but in poorly differentiated and neurally invasive tumors, it might play rather a major role in later stages of tumorigenesis and metastasis [52]. Consistent with these findings, SOX2 has been implicated in Mesenchymal-to-Epithelial Transition (MET), the reverse process of EMT, as *SOX2* KD in colorectal cancer cells alters the expression of key genes involved in EMT, leading to increased E-cadherin and decreased Vimentin expression [53]. Furthermore, *de novo* SOX2 expression in PDAC cells is sufficient to promote self-renewal, dedifferentiation, and affects stemness characteristics *via* modulation of specific cell cycle regulatory genes and EMT driver genes [54]. In line with these findings, our group could provide evidence that expression of Nestin and SOX2 is associated with distinct EMT phenotypes in PDAC cells [43]. Characterization of isolated and expanded CSC variants (so called Holoclone cells) from the PDAC cell lines Panc1 and Panc89 revealed that Nestin is associated with a mesenchymal-like phenotype of Panc1 cell variants, while SOX2 is related to the epithelial phenotype of Panc89 cells [43, 47, 55]. Furthermore, these distinct CSC-EMT phenotypes are associated with different functional properties like colony formation ability, migration and invasion potential, response to chemotherapy and tumorigenicity. Following these results, the present study aimed to elucidate the impact of Nestin and SOX2 on the different CSC-EMT PDAC cell variants to improve our understanding of the regulation of PDAC cell plasticity.

## Materials and methods

### Cell Lines and Cell Culture

As a model for mesenchymal-like PDAC cells, the human cell line Panc1 was used (ATCC, Manassas, Virginia, US) originating from a primary tumor of a male PDAC patient. As a model for PDAC cells with an epithelial phenotype, the human cell line Panc89 (kindly provided by Prof. T. Okabe, University of Tokyo, JP) originating from a lymph node metastasis of a male PDAC patient, was used. Holoclone cells of both cell lines were isolated and expanded *via* single-cell cloning as described [43]. All cell variants were cultivated in Panc-medium (RPMI 1640 supplemented with 10% FCS, 1% L glutamine, and 1% sodium pyruvate (Biochrom, Berlin, DE)). Incubation of cells was conducted at 37°C with 5% CO_2_ and 86% humidity.

## siRNA-mediated Knockdown of CSC Markers

To investigate the functional impact of Nestin and SOX2, PDAC cell variants were subjected to siRNA-mediated KD of *NES* and/or *SOX2*. Gene silencer reagents are scrambled and usually consist of a mixture of 3 to 5 target-specific 19–25 nucleotide long sequences. KD of *NES* was performed in Panc1 cell variants and KD of *SOX2* was performed in Panc89 cell variants. Double KD of *NES*/*SOX2* was performed in all Panc1 and Panc89 cell variants for RT-qPCR, immunofluorescence staining (IFS), viable cell count analysis, colony formation assay (CFA), as well as in Panc1 and Panc89 Holoclone cells for Western Blot analysis. For this purpose, 5 × 10^4^ cells/ well were seeded in 12-well plates and cultivated in 1 ml Panc-medium. After 24 h, the cells were transfected with 100 µl OPTI-MEM^®^ (Gibco *via* Thermo Scientific, Schwerte, DE) containing 4 µl HiPerfect^®^ (Qiagen GmbH, Hilden, DE) and 4 µl (0.12 µM) of the respective specific siRNA or a control siRNA (CTRLsi) (Table 1). The double KD of *NES* and *SOX2* in Panc1 and Panc89 cell variants was performed with 200 µl OPTI-MEM^®^ containing 8 µl HiPerfect^®^ and 4 µl (0.12 µM) of each specific siRNA or CTRLsi. The transfectant was mixed and incubated for 10 min at RT before adding it to the cells. CTRLsi A was used for Panc1 cell variants, and CTRLsi B was used for Panc89 cell variants. For the double KD, both CTRLsi A and B were used. Transfection was performed for 72 h, until cells were subjected to different analyses. Success and stability of the siRNA mediated KD was checked regularly by RT-qPCR and IFS.

**Table 1:** Small interfering RNA (siRNA) used for knockdown of Nestin and SOX2

siRNA	Stock [µM]	Manufacturer
Control siRNA-A (sc-37007)	10	Santa Cruz Biotechnology, Texas, US
Control siRNA-B (sc-44230)	10	Santa Cruz Biotechnology, Texas, US
Nestin siRNA (human) (sc-36032)	10	Santa Cruz Biotechnology, Texas, US
SOX2 siRNA (human) (sc-38408)	10	Santa Cruz Biotechnology, Texas, US

## RNA Isolation and RT-qPCR

Total RNA was isolated using the total RNA kit peqGOLD (PeqLab, Erlangen, DE) and subjected to reverse transcription according to manufacturer’s instructions (Fermentas *via* Thermo Fisher Scientific, Darmstadt, DE). RT-qPCR analysis was performed in duplicates on a LightCycler 480 (Roche, Basel, CH) for a maximum of 50–60 cycles ending with a melting curve analysis for primer quality control. Primers used (Eurofins, Ebersberg DE; RealTime Primers *via* Biomol, Hamburg, DE), primer sequences, and annealing-temperatures are listed in Table 2. For relative quantification of RNA levels, CT-values of genes of interest were normalized to the respective CT-value for the reference gene GAPDH.

**Table 2:** Target genes, primer sequences, and annealing temperatures used for RT-PCR

Target	5`- 3` Sequence	Annealing [C°]
*CDH1* (E-cadherin)^**^	fw - TGCTCTTGCTGTTTCTTCGG rv - TGCCCCATTCGTTCAAGTAG	55
*GAPDH* ^*^	fw - TCCATGACAACTTTGGTATCGTGG rv - GACGCCTGCTTCACCACCTTCT	58
*L1CAM* ^**^	fw - GAACTGGATGTGGTGGAGAG rv - GAGGGTGGTAGAGGTCTGGT	58
*NES* (Nestin)^*^	fw - GAAACAGCCATAGAGGGCAAA rv - TGGTTTTCCAGAGTCTTCAGTGA	58
*OVOL2* (ZNF339)^**^	fw - GGGACAAGCTCTACGTCTGC rv - GTCTGTCCTCCCCTTCCTTC	58
*SOX2* ^*^	fw - TCCCATCACCCACAGCAAATGA rv - TTTCTTGTCGGCATCGCGGTTT	58
*VIM* (Vimentin)^**^	fw - TCCAAGTTTGCTGACCTCTC rv - TCAACGGCAAAGTTCTCTTC	58
*ZEB*1^*^	fw - TCCATGCTTAAGAGCGCTAGCT rv - ACCGTAGTTGAGTAGGTGTATGCCA	61
*ZEB2* ^**^	fw - CACATCAGCAGCAAGAAATG rv - AAACCCGTGTGTAGCCATAA	58

* = Primers purchased from Eurofins Genomics GmbH (Ebersberg, DE). Stocks of forward (fw) and reverse (rv) diluted at 1 pM/µl in nuclease-free ddH_2_O

** = Primers purchased from RealTime Primers (*via* Biomol, Hamburg, DE). Provided as stocks of 50 µM primer mix (containing fw and rv primer) in nuclease-free ddH_2_O supplemented with 10 mM Tris-HCL and 0.1 mM EDTA (pH 7.5)

## Immunofluorescence Staining

To confirm the siRNA-mediated KD of *NES* in Panc1 cell variants, KD of *SOX2* in Panc89 cell variants and double KD of *NES/SOX2* in both Panc1 and Panc89 cell variants on protein level, an IFS was performed using an anti-Nestin antibody (clone 10C2, Thermo Scientific, Schwerte, DE) and an anti-SOX2 antibody (clone D6D9, Cell Signaling Technology, Danvers, MA, US). For this purpose, 2 × 10^4^ cells/well were seeded on glass coverslips in 12-well plates. After 24 h, cells were transfected as described above. After 72 h, transfectant containing medium was removed and coverslips were washed with PBS. Fixation of the cells was performed by incubation with 4% PFA for 15 min at RT. Next, the coverslips were washed with PBS and incubated with ice-cold methanol for 10 min at -20 °C for permeabilization of the cells. Afterwards, the coverslips were washed with PBS and cells were blocked with 4% bovine serum albumin (BSA) diluted in 0.3% TritonX-100 in PBS for 1 h at RT. After washing with 0.3% TritonX-100 in PBS, incubation with the primary antibodies was performed (see below).

Incubation of every antibody and Hoechst 33258 (Merck Millipore, Darmstadt, DE) was performed in 1% BSA diluted in 0.3% TritonX-100 in PBS and in a humidity chamber. Incubation of primary antibodies was performed overnight (ON) at 4 °C, whereas incubation of the secondary antibodies was performed for 1 h at RT. Both, the anti-SOX2 antibody and its corresponding isotype CTRL (rabbit IgG, Bio-Techne, MN, US) were used at a concentration of 25 µg/ml. The anti-Nestin antibody and the corresponding isotype CTRL (mouse IgG1, R&D Systems GmbH, Wiesbaden, DE) were used at a concentration of 5 µg/ml. Secondary antibodies (AlexaFluor goat-anti-rabbit 647 and AlexaFluor goat-anti-mouse 488, Invitrogen, Carlsbad, US) both were used at a concentration of 2 µg/ml. Dilution of secondary antibodies was supplemented with 2 µg/ml Hoechst 33258 for nuclei staining. Between incubation with primary and secondary antibodies a washing step with 0.3% TritonX-100 in PBS was performed. After washing with PBS and dH_2_O, coverslips were sealed with Fluor Safe Reagent (Electron Microscopy Sciences, Hatfield, PA). Image acquisition was performed with the Lionheart FX Automated Microscope and related software (Gen5 Data Analysis Software (3.10), Bio-Tek, Bad Friedrichshall, DE).

## Viable Cell Count Analyses

For the viable cell count analyses 5 × 10^4^ cells/well were seeded in 12-well plates and after 24 h, cells were transfected as described above. After 72 h transfection, the transfectant containing medium was removed, cells were washed with PBS and detached from the plate. After centrifugation, the supernatant was discarded and 1 ml fresh Panc-medium was added. The cells were mixed with Trypan Blue to reach a final concentration of 0.01% and pipetted to a 96-well plate. The plate was centrifuged at 30×g for 1 min and automatically imaged with NYONE^®^ Scientific (SYNENTEC GmbH, Elmshorn, DE). The Trypan Blue application of YT-SOFTWARE^®^ (SYNENTEC GmbH, Elmshorn, DE) was used to determine the viable cell density.

### Colony Formation Assay

Panc1 and Panc89 cell variants were seeded for transfection as described above. After 72 h, cells were detached by trypsinization and re-seeded in 2 ml Panc-medium in 12-well plates. For Panc1 cell variants 400 cells/well and for Panc89 cell variants 200 cells/well were seeded. Colony formation was monitored for 6–10 days and the experiment was terminated by washing the cells with PBS and fixation with 0.5 ml 4% PFA per well for 15 min at RT. Afterwards, fixed cells were washed twice with dH_2_O, stained with 0.1% crystal violet (Merck Millipore, Darmstadt, DE) for 1 h, washed in dH_2_O and air-dried at RT. Only colonies containing more than 50 cells were counted and their morphology regarding Holo-, Mero- and Paraclones was determined. Holoclones, which are associated with a strong CSC potential, consisted of tightly, homogenously clustered cells with a regular borderline. Paraclones, which are described to be the more differentiated cancer cell population, consisted of dispersed, larger cells with an irregular boundary [55]. Meroclones form an intermediate state between Holo- and Paraclones [55].

## Migration Assay

Panc1 and Panc89 cell variants were transfected as described above. After 72 h, cells were detached and analyzed for cell migration, which was conducted in 24-well plates (Greiner Bio-One GmbH, Frickenhausen, DE) with Ibidi^®^ 2-well culture inserts (Ibidi GmbH, Gräfelfing, DE). Transfected Panc1 and Panc89 cell variants were seeded with 4 × 10^4^ cells and 3 × 10^4^ cells, respectively, in 75 µl Panc-medium in each cavity of the Ibidi^®^ insert. After 24 to 48 h, when a confluent cell layer was detectable, the Ibidi^®^ inserts were removed and the cells were washed with prewarmed PBS. Afterwards, fresh Panc-medium without FCS was added to the cells. Cell migration of Panc1 and Panc89 cell variants was monitored after 24 h, using the NYONE^®^ Scientific imaging device (SYNENTEC GmbH, Elmshorn, DE). The Wound Healing application of the YT-SOFTWARE^®^ (SYNENTEC GmbH, Elmshorn, DE) was used to determine the confluence on the original gap.

## Invasion Assay

Panc1 and Panc89 cell variants were transfected as described above. After 72 h, the cells were detached and 1 × 10^4^ cells (Panc1 cell variants) and 1.5 × 10^4^ cells (Panc89 cell variants) were seeded in 200 µl Panc-medium in each well of a clear 96-well ultra-low-attachment plate (faCellitate, Mannheim, DE). Afterwards, the plate was centrifuged at 300 ×g at RT for 5 min and incubated at 37 °C, 5% CO_2_ and 85% relative humidity for 24 h to allow spheroid formation. After 24 h, 176 µl cell culture medium were removed from each well and the plate was centrifuged again at 300 ×g and 4°C for 30 s. The plate was then put on ice and the spheroids formed were covered with 76 µl of Matrigel^®^ (Corning GmbH, Kaiserslautern, DE). Finally, the plate was incubated at 37 °C, 5% CO_2_ and 85% relative humidity for 30 min before the first measurement with the NYONE^®^ Scientific imaging device (SYNENTEC GmbH, Elmshorn, DE) and the related Spheroid Quantification application of the YT-SOFTWARE^®^ (SYNENTEC GmbH, Elmshorn, DE) was performed. Spheroids were imaged every 24 h for 72 h and 120 h for Panc1 and Panc89 cell variants, respectively. Spheroid invasion was determined by manually analyzing the number of invasive fronts and measuring the distance of each invasive front from the spheroid border using the measurement mode of the YT-SOFTWARE^®^ (SYNENTEC GmbH, Elmshorn, DE).

### Assessment of Treatment Responses Towards Cytostatic Drugs

Panc1 and Panc89 cell variants were transfected as described above. After 72 h, cells were detached and 1 × 10^3^ cells/well were seeded in 200 µl Panc-medium in clear flat-bottom 96-well plates. After 24 h, cells were washed with Panc-medium and both transfection conditions (CTRLsi, specific siRNA) of either cell variant were left untreated or treated with Gemcitabine, 5-FU or Oxaliplatin in the concentrations shown in Table 3 in duplicates. After 72 h, an endpoint analysis was performed by staining with 2 µg/ml Hoechst 33342 (Thermo Scientific, Schwerte, DE) and 20 µg/ml propidium iodide (Merck Millipore, Darmstadt, DE). The plates were imaged using the NYONE^®^ Scientific imaging device and the Virtual Cytoplasm 1 F application of the YT-SOFTWARE^®^ (SYNENTEC GmbH, Elmshorn, DE) to determine the number of total cells (Hoechst-positive) and dead cells (Hoechst and propidium iodide-double positive).

**Table 3:** Concentrations of the cytostatic drugs (Gemcitabine, 5-Fluorouracil, Oxaliplatin) used for treatment of Panc1 and Panc89 cell variants

Cell variant	Gemcitabine	5-Fluorouracil	Oxaliplatin
Panc1 cell variants	0.0014 µM	1.368 µM	0.3337 µM
Panc89 cell variants	0.0034 µM	0.2629 µM	0.7761 µM

### Statistical Analysis

The statistical evaluation was carried out using GraphPad Prism Software (Version 10.4.0 (Docmatics, San Diego, California, US). All data sets were tested for normal distribution using Shapiro-Wilk test.

Parametric data including multiple groups were tested by one-way analysis of variance (one-way ANOVA) for statistical significance. Non-parametrical datasets of multiple groups were analyzed with Kruskal-Wallis one-way ANOVA on ranks test. Statistically significant differences between the groups were assumed at p-values ≤ 0.05 according to Student-Newman-Keuls method (parametric data) and Dunn’s method (non-Parametric data), respectively.

Graphs of parametric data were presented by mean with standard deviation or mean with standard error of means (depending on technical and biological replicates), while graphs of non-parametric data were presented by median with (interquartile) range. Students t-test was used to examine two samples of normally distributed data. Grouped data sets were analyzed using two-way-ANOVA and Tukey´s multiple comparisons test. All data sets were examined using the appropriate statistical tests. Non-significant data are not presented in the graphs. Significances between groups are shown as zig-zag line with asterisk. Significances of normalized data of controls (which are not shown as an additional bar) and tested groups, are presented with a line and asterisk above the test group. Statistical significance is defined at a p-value of ≤ 0.033 and are indicated by asterisks (*p* ≤ 0.033= *, *p* ≤ 0.002= **, *p* ≤ 0.001= ***) in the graphs.

## Results

**siRNA-mediated KD leads to a significant reduction of** ***NES*** **expression in Panc1 cell variants and** ***SOX2*** **expression in Panc89 cell variants**.

**Fig. 1:**
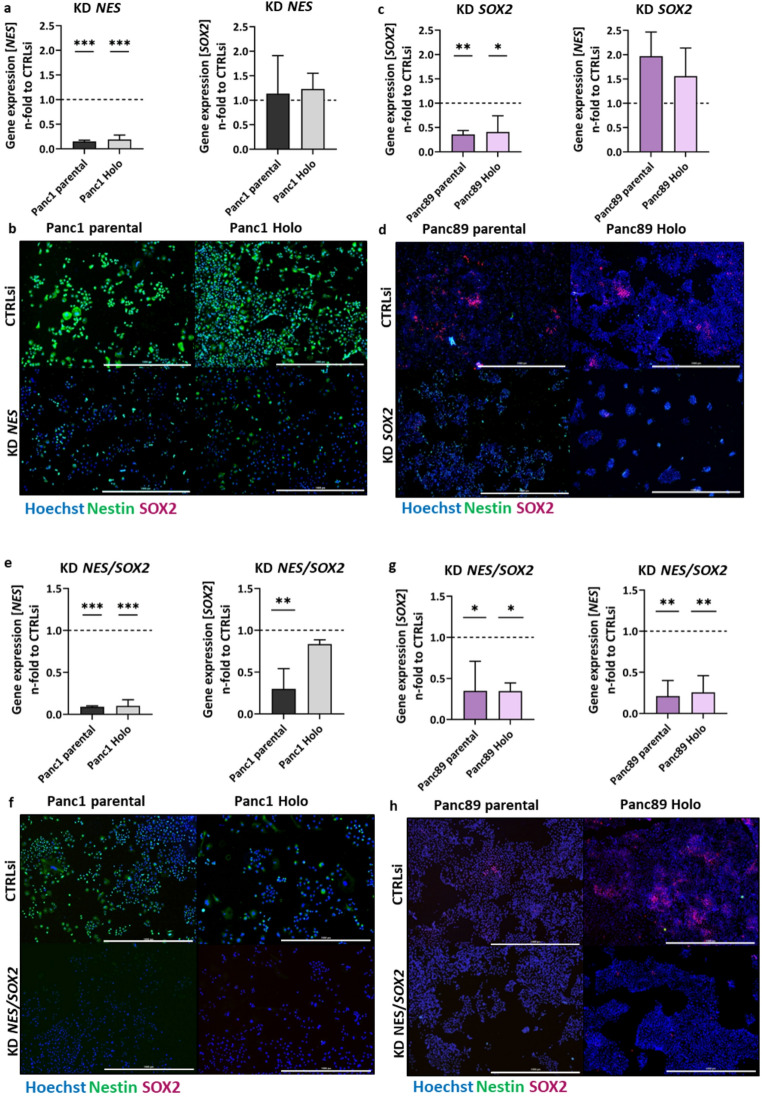
**Confirmation of siRNA-mediated KD of** ***NES*** **expression in Panc1 cell variants and** ***SOX2*** **expression in Panc89 cell variants.** 5 × 10^4^ cells were subjected to siRNA-mediated KD of *NES* in Panc1 cell variants, *SOX2* in Panc89 cell variants and double KD of *NES/SOX2* in all cell variants or to CTRLsi transfection for 72 h. Afterward, the KD was confirmed by RT-qPCR (a, c, e, g) and IFS (b, d, f, h). Every analysis was performed with *n* = 3 independent experiments. Gene expression data of *NES* and *SOX2* (a, c, e, g) are normalized to CTRLsi conditions. All data sets were tested for normal distribution using Shapiro-Wilk test, followed by one-way ANOVA for statistical significance. Significances are indicated by asterisk: *p* ≤ 0.033 = *, *p* ≤ 0.002 = **, *p* ≤ 0.001 = ***. Data are presented by mean with SD. Representative IFS images from *n* = 3 independent experiments are shown (b, d, f, h). Scale bars IFS = 1000 μm. (KD = knockdown; RT-qPCR = real time quantitative polymerase chain reaction, IFS = immunofluorescence staining, CTRLsi = control siRNA; SD = standard deviation; Holo = Holoclone)

To determine the success of the siRNA-mediated KD of *NES* in Panc1 cell variants and *SOX2* in Panc89 cell variants, parental and Holoclone cells of either cell line were transfected for 72 h followed by gene expression (Fig. 1a, c) and protein level (Fig. 1b, d) analysis *via* RT-qPCR and IFS, respectively.

In both Panc1 cell variants a significant reduction of *NES* expression by 70–80% was observed after KD of *NES*, while the expression of *SOX2* was not affected (Fig. 1a). In line with these findings, Nestin protein levels were decreased in parental Panc1 and Panc1 Holoclone cells by about 80% while SOX2 was not visible in both cell variants under either condition (Fig. 1b). Gene expression analysis after *SOX2* KD in both Panc89 cell variants showed a significant reduction of *SOX2* expression by 60–70% together with an 1.5-2.0 -fold increase of *NES* expression compared to CTRLsi condition (Fig. 1c). Protein analysis of Panc89 cell variants after KD of *SOX2* revealed an even complete loss of SOX2 together with slightly increased Nestin levels in parental Panc89 cells (Fig. 1c, d).

To further explore the impact of both CSC markers in PDAC cells, a simultaneous KD of *NES* and *SOX2* was performed in Panc1 and Panc89 cell variants (Fig. 1e-h). Gene expression analysis after KD of *NES/SOX2* showed a significant decrease of *NES* expression in parental Panc1 and Panc1 Holoclone cells and a significant reduction of *SOX2* in parental Panc1 cells, while the decrease in *SOX2* expression in Panc1 Holoclone cells was less pronounced (Fig. 1e). On protein level, both Panc1 cell variants showed considerable Nestin staining under CTRLsi and a complete loss of Nestin after KD of *NES/SOX2*, while SOX2 was again not detectable under both conditions (Fig. 1f). KD of *NES/SOX2* in parental Panc89 and Panc89 Holoclone cells led to a significant reduction of *NES* and *SOX2* in both Panc89 cell variants (Fig. 1g). On protein level, parental Panc89 and Panc89 Holoclone cells exhibited SOX2 staining and showed a complete loss of SOX2 protein levels after KD of *NES/SOX2*, while Nestin was not detectable under both conditions (Fig. 1h). Overall, a significant siRNA-mediated decrease of Nestin and SOX2 levels after single KD, respectively, and after double KD could be confirmed in Panc1 and Panc89 cell variants, respectively.

**KD of** ***NES*** **in Panc1 and KD of** ***SOX2*** **Panc89 cell variants slightly modulate gene expression of EMT marker and plasticity modulators**.

Next, the impact of the siRNA-mediated single or double KD of *NES* and *SOX2* in Panc1 and Panc89 cell variants on EMT marker expression was determined *via* RT-qPCR. For this purpose, an established panel of EMT markers (*CDH1*, *L1CAM*, *VIM*) and plasticity modulators (*ZEB1*, *ZEB2*,* OVOL2*) was analyzed [43].

**Fig. 2:**
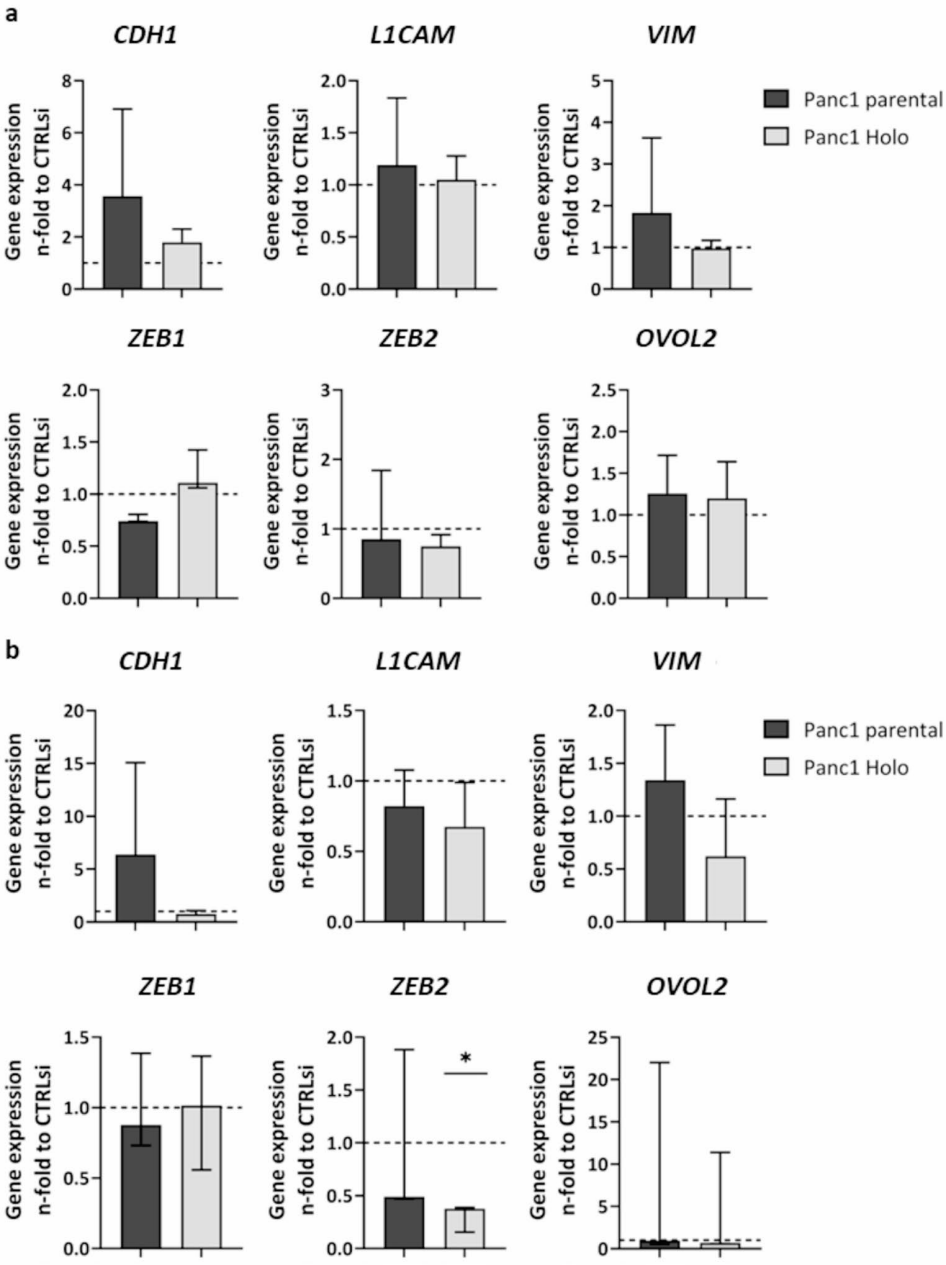

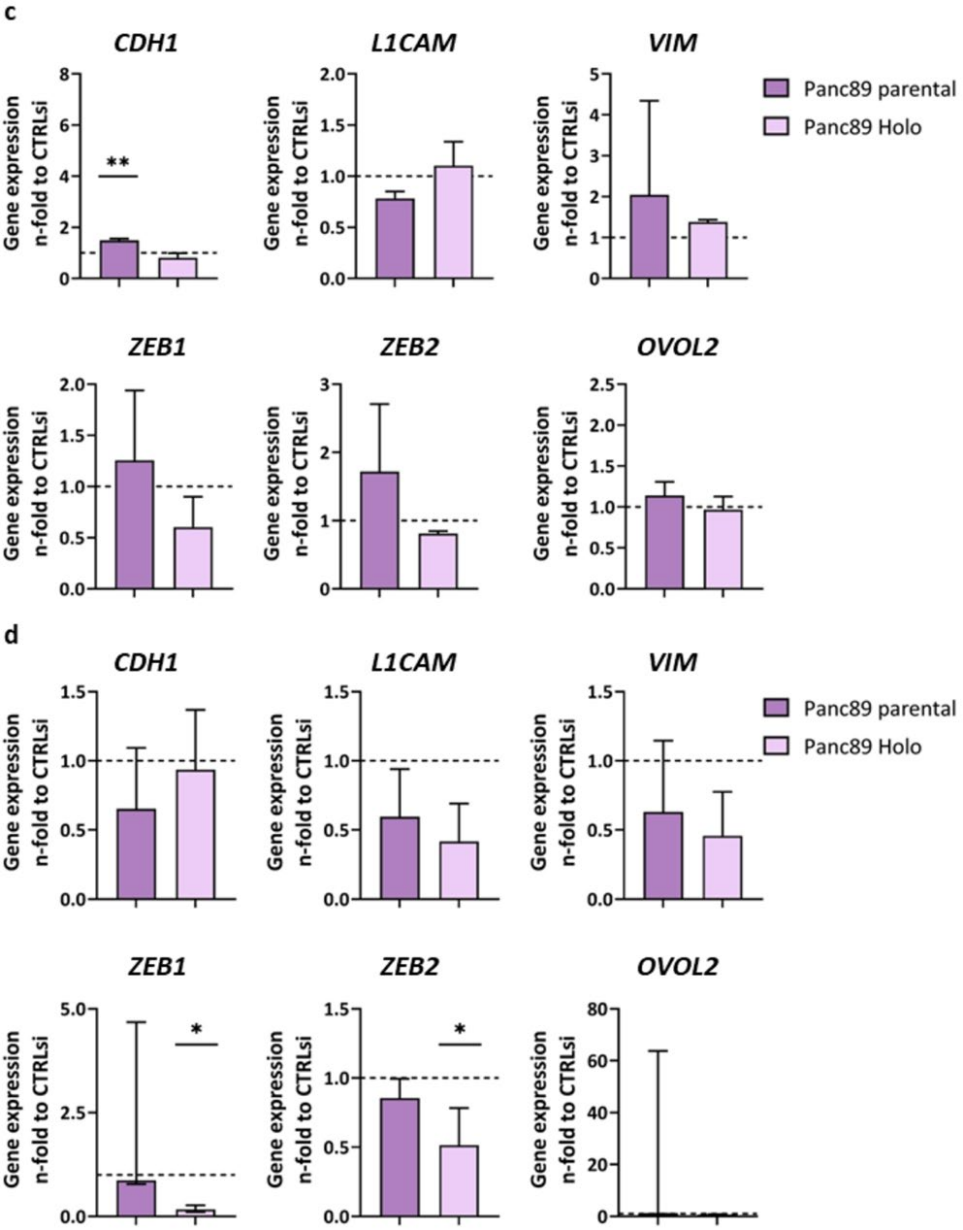
**Gene expression analysis of EMT markers and plasticity modulators after single and double KD of** ***NES*** **and** ***SOX2*** **in Panc1 and Panc89 cell variants.** 5 × 10^4^ cells were subjected to siRNA-mediated KD of *NES* in Panc1 cell variants (a), *SOX2* in Panc89 cell variants (c), double KD of *NES/SOX2* in Panc1 cell variants (b) and Panc89 cell variants (d) or to CTRLsi transfection (a-d) for 72 h. Afterward, gene expression of EMT markers (a-d top: *CDH1*, *L1CAM*, *VIM*) and plasticity modulators (a-d bottom: *ZEB1*, *ZEB2*, *OVOL2*) was determined *via* RT-qPCR. Analysis was performed with *n* = 3 independent experiments. Gene expression data of all genes of interest were first normalized to the reference gene GAPDH and then normalized to CTRLsi conditions. All data sets were tested for normal distribution using Shapiro-Wilk test. Parametric data were tested by one-way ANOVA and are presented by mean with SD. Non-parametric data were tested by Kruskal-Wallis one-way ANOVA on ranks and are presented as median with interquartile range. Significances are indicated by asterisk: *p* ≤ 0.033 = *, *p* ≤ 0.002 = **, *p* ≤ 0.001 = ***. (CSC = cancer stem cell; EMT = Epithelial-to-Mesenchymal Transition; KD = knockdown; RT-qPCR = real time quantitative polymerase chain reaction; CTRLsi = control siRNA; SD = standard deviation; Holo = Holoclone)

Investigating the impact of *NES* or *SOX2* KD on gene expression of EMT markers and plasticity modulators revealed a considerable impact on the epithelial marker E-cadherin (*CDH1*) after KD of *NES* in parental Panc1 and Panc1 Holoclone cells exhibiting a 3.5- and 1.5-fold increased expression, respectively, indicating a switch from a mesenchymal to a more epithelial phenotype (Fig. 2a). In parental Panc1 cells, this effect was even strengthened by the double KD of *NES/SOX2* leading to a more than 5-fold increased expression of *CDH1*, while Panc1 Holoclone cells were unaffected (Fig. 2b). Expression of the mesenchymal markers *L1CAM* and Vimentin (*VIM*) was marginally impacted by single KD of *NES* (Fig. 2a), while simultaneous KD of *NES/SOX2* forced a decrease in *L1CAM* expression in both Panc1 cell variants together with reduced *VIM* expression in Panc1 Holoclone cells (Fig. 2b).

Gene expression of the plasticity modulators *ZEB1*, *ZEB2* and *OVOL2* after single and double KD of *NES* and *SOX2* in Panc1 cell variants revealed, that single KD of *NES* (Fig. 2a) and double KD of *NES/SOX2* (Fig. 2b) led to a slight decrease of *ZEB1* in parental Panc1 cells, while *ZEB1* expression in Panc1 Holoclone cells was hardly affected. *ZEB2* expression was marginally decreased in both Panc1 cell variants after single *NES* KD (Fig. 2a), an effect which was further intensified after double KD of *NES/SOX2* being more pronounced in Panc1 Holoclone cells (Fig. 2b). *OVOL2* expression was almost unaffected after single *NES* KD and double KD of *NES/SOX2* in both Panc1 cell variants (Fig. 2a, b).

Single KD of *SOX2* in Panc89 cell variants also led to a significant increase of *CDH1* in parental Panc89 cells (Fig. 2c), while KD of *NES/SOX2* reversed this effect leading to a reduction in *CDH1* expression in parental Panc89 cells (Fig. 2d). *CDH1* expression in Panc89 Holoclone cells remained almost unaffected after both, *SOX2* KD and KD of *NES/SOX2* (Fig. 2c, d).

KD of *SOX2* in Panc89 cell variants led to slightly increased *ZEB1* and *ZEB2* expression in parental Panc89 cells (Fig. 2c), while KD of *NES/SOX2* slightly reduced the expression (Fig. 2d) as observed in Panc1 cell variants (Fig. 2b). In Panc89 Holoclone cells the expression of *ZEB1* and *ZEB2* was slightly decreased after *SOX2* KD (Fig. 2c), an effect that was further strengthened after double KD of *NES/SOX2* (Fig. 2d). As observed in Panc1 cell variants, *OVOL2* expression was almost unaffected after single *SOX2* KD as well as after KD of *NES/SOX2* in both Panc89 cell variants (Fig. 2c, d). Overall, gene expression analysis of EMT markers revealed only marginal effects after KD of *NES* in Panc1 cell variants and KD of *SOX2* in Panc89 cell variants with the strongest effect on *CDH1* expression in parental cell variants after either KD, showing a possible effect towards a more epithelial phenotype in these cells. Furthermore, double KD of *NES/SOX2* exerted decreasing effects on EMT markers and plasticity modulators, especially in the Holoclone populations. Since single and double KD led to the most prominent alterations in the respective Holoclone populations, Western Blot analysis was performed to validate these findings. However, since KD mediated changes were not visible on the protein level in Panc1 and Panc89 holoclone cells (**Supplementary Fig. 1**), the impact of Nestin and SOX2 on EMT characteristics in PDAC cells analyzed seems to be marginal.

**KD of** ***NES*** **in Panc1 cell variants and KD of** ***SOX2*** **in Panc89 cell variants marginally impact cell growth but significantly decreases self-renewing properties**.

**Fig. 3:**
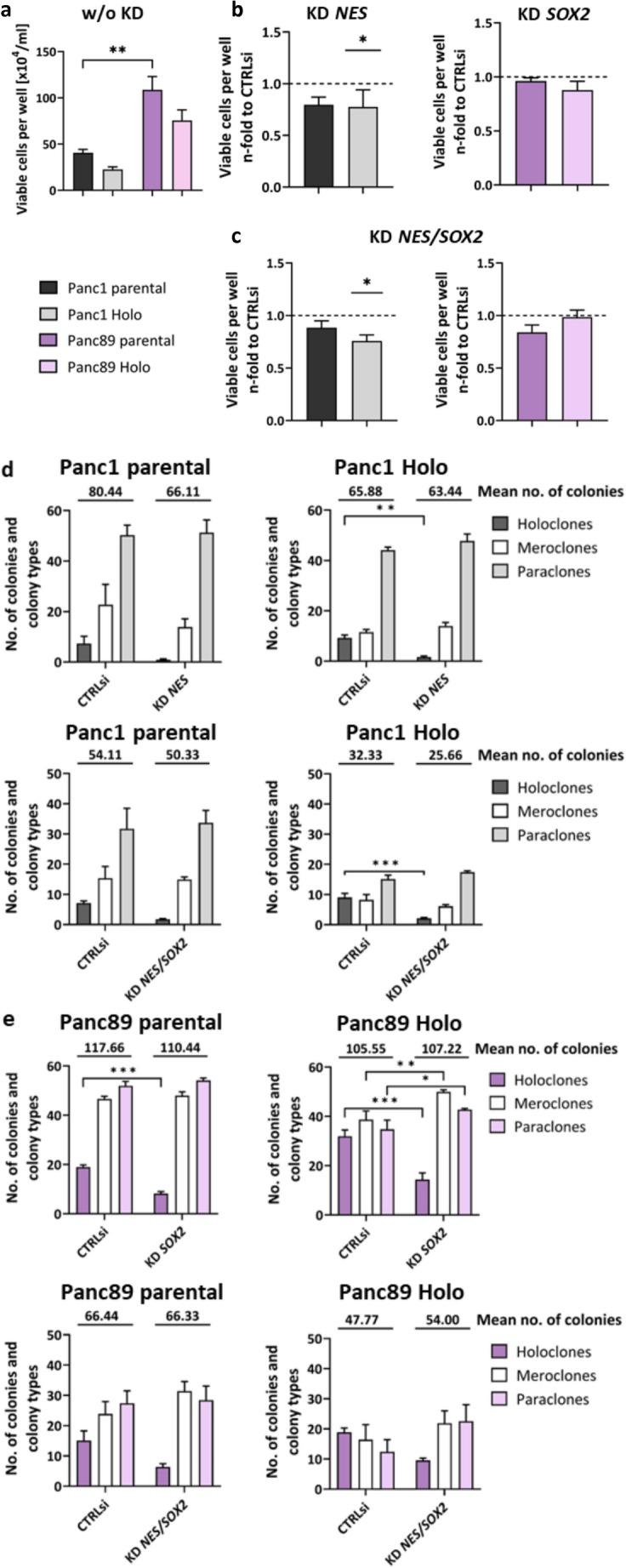
**KD of** ***NES*** **in Panc1 cell variants and KD of** ***SOX2*** **in Panc89 cell variants marginally impact cell growth but significantly decrease self-renewing properties.** 5 × 10^4^ cells were either left untreated (a) or were subjected to siRNA-mediated KD of *NES* in Panc1 cell variants (b, d), *SOX2* in Panc89 cell variants (c, e), double KD of *NES/SOX2* in all cell variants (c-e) or to CTRLsi transfection (b-e) for 72 h. Afterward, the number of viable cells was determined by Trypan blue staining (a-c) and cells were re-seeded with 400 cells/well for Panc1 cell variants or 200 cells/well for Panc89 cell variants for CFA in 12-well plates (d-e). The number of viable cells under KD of *NES* (b), *SOX2* (b) or *NES*/*SOX2* (c) was normalized to CTRLsi conditions. Data were tested for normal distribution by Shapiro-Wilk analysis, followed by one-way ANOVA and Tukey´s multiple comparison test. d-e) CFAs were monitored for 6–10 d, fixed with PFA and stained with crystal violet. CFA data were analyzed by two-way ANOVA and Tukey´s multiple comparison. Every analysis was performed with *n* = 3 independent experiments plus technical replicates and data are shown as mean with SEM. Significances are indicated by asterisk: *p* ≤ 0.033 = *, *p* ≤ 0.002 = **, *p* ≤ 0.001 = ***. (KD = knockdown; CTRLsi = control siRNA; CFA = colony formation assay; SEM = standard error of means; Holo = Holoclone; PFA = paraformaldehyde; SEM = standard error of means; Holo = Holoclone; No. = Number; w/o KD = without knockdown)

To determine the impact of *NES* KD in Panc1 cell variants, *SOX2* KD in Panc89 cell variants as well as KD of *NES/SOX2* in all four PDAC cell variants on functional properties, a viable cell count analysis and CFA were performed (Fig. 3).

Overall, mesenchymal Panc1 cell variants showed a slower cell growth compared to epithelial Panc89 cell variants (Fig. 3a), being in line with previous findings [43].

KD of *NES* in Panc1 cell variants decreased the viable cell number compared to CTRLsi treated cells, with a significantly lower viable cell number of Panc1 Holoclone cells (Fig. 3b). KD of *SOX2* in Panc89 cell variants hardly affected both Panc89 cell variants, only a slight decrease in the number of viable cells was observed in Panc89 Holoclone cells compared to CTRLsi conditions (Fig. 3b). In Panc1 cell variants, double KD of *NES/SOX2* led to similar effects compared to single KD of *NES*, with a significantly reduced number of viable Panc1 Holoclone cells (Fig. 3c). In Panc89 cell variants, the double *NES/SOX2* KD only marginally impacted the number of viable cells, albeit the effect was reversed to that of single *SOX2* KD (Fig. 3b).

The CFA demonstrated that the mean number of total colonies formed marginally decreased under either single KD of *NES* and double *NES*/*SOX2* KD in Panc1 cell variants, compared to CTRLsi conditions (Fig. 3d). A more detailed analysis of the colonies formed revealed that all Panc1 cell variants showed a strong decrease of Holoclone colonies after single KD of *NES*, but which was not further intensified by double *NES/SOX2* KD (Fig. 3d). Similar results were observed after single *SOX2* KD and double *NES/SOX2* KD in both Panc89 cell variants (Fig. 3e). Only marginal effects of either KD on the total number of colonies formed by parental Panc89 cells as well as Panc89 Holoclone cells were detected, while formation of Holoclones was clearly reduced after single *SOX2* KD and double KD, respectively (Fig. 3e). The fact that colony formation of all PDAC cell variants was generally lower after double KD compared to either single KD can be attributed to transfection conditions as the number of total colonies was also reduced under CTRLsi conditions (Fig. 3d, e).

Overall, this data suggests that KD of *NES* and *SOX2* only marginally impact cell growth and cell viability in Panc1 and Panc89 cell variants, respectively, but clearly impair the self-renewal abilities reflected by the diminished numbers of Holoclone colonies after either CSC marker KD in all analyzed PDAC cell variants. The fact that the double KD of both CSC markers did not further impair colony formation in all PDAC cell variants indicates that self-renewal capacity of PDAC cells can be maintained in different ways being dependent on the cellular context (e.g. expression of CSC markers).

**KD of** ***NES*** **in Panc1 cell variants and KD of** ***SOX2*** **in Panc89 cell variants marginally affect migration and invasion properties**.

**Fig. 4:**
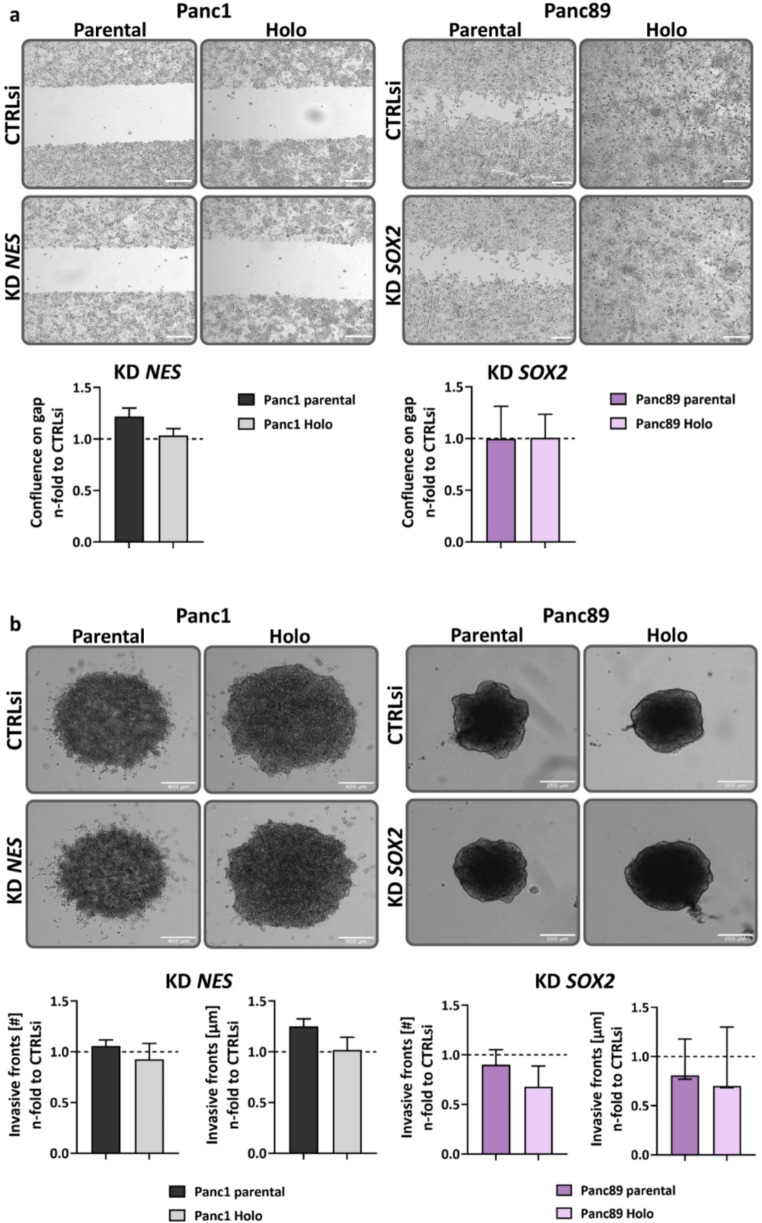
**siRNA-mediated KD of** ***NES*** **in Panc1 and** ***SOX2*** **in Panc89 cell variants marginally impacts migration and invasion properties.** 5 × 10^4^ cells were subjected to siRNA-mediated KD of *NES* in Panc1 and *SOX2* Panc89 cell variants or to CTRLsi transfection for 72 h. Afterward, transfected cells were detached and seeded in (a) Ibidi^®^ 2-well culture inserts (4 × 10^4^ for Panc1; 3 × 10^4^ for Panc89 cell variants) and migration was analyzed after 24 h for Panc1 and Panc89 cell variants, or in (b) 96-well ULA plates for spheroid formation (1 × 10^4^ for Panc1 cell variants; 1.5 × 10^4^ for Panc89 cell variants) and determination of invasion properties. Cell invasion (number of invasive fronts [#]; invasion distance [µm]) was analyzed after 72 and 120 h for Panc1 and Panc89 cell variants, respectively. Every analysis was performed with *n* = 3 independent experiments plus technical replicates and *NES* and *SOX2* KD values, respectively, were normalized to CTRLsi conditions. All data sets were tested for normal distribution using Shapiro-Wilk test. Parametric data were tested by one-way ANOVA with Tukey´s multiple comparison and are presented by mean with SEM. Non-parametric data were tested by Kruskal-Wallis one-way ANOVA on ranks and are presented as median with interquartile range. Representative images from *n* = 3 independent experiments are shown (a, b; top). Scale bar a) = 200 μm, b) left = 400 μm, b) right = 200 μm. (KD = knockdown; CTRLsi = control siRNA; ULA = ultra-low-attachment plate; SEM = standard error of means; Holo = Holoclone)

Next, the effects of KD of *NES* in Panc1 and *SOX2* in Panc89 cell variants on migration and invasion properties were determined (Fig. 4). In line with previous findings, Panc89 cell variants exhibited an increased migratory potential compared to Panc1 cell variants [43]. As shown in Fig. 4a, KD of *NES* in parental Panc1 cells slightly increased cell migration compared to CTRLsi conditions, while migration of Panc1 Holoclone cells was not affected. Moreover, *SOX2* KD did not impact migration abilities of either Panc89 cell variant (Fig. 4a).

In line with the EMT phenotype, Panc1 cell variants showed a pronounced cell invasion in a mesenchymal cell manner, while Panc89 cell variants used an epithelial cluster-like invasion mode occurring over a longer period of time compared to the mesenchymal-like invasion [43]. Parental Panc1 cells showed a slightly increased distance of invasive fronts [µm], while the number of invasive fronts [#] was unaffected by KD of *NES* (Fig. 4b). Both, number of invasive fronts [#] and covered distance of invasive fronts [µm] remained unaffected in Panc1 Holoclone cells after *NES* KD (Fig. 4b). In contrast, KD of *SOX2* in parental Panc89 as well as Panc89 Holoclone cells led to a decreased invasion by trend with respect to both number of invasive fronts [#] and covered distance of invasive fronts [µm] (Fig. 4b).

Overall, KD of *NES* in Panc1 cell variants and *SOX2* in Panc89 cell variants only marginally impact cell migration and invasion, suggesting that the CSC markers Nestin and SOX2 alone are not key players in the regulation of migration and invasion of these PDAC cell variants.

**KD of** ***NES*** **in Panc1 cell variants and KD of** ***SOX2*** **in Panc89 cell variants only marginally impact the response to cytostatic drugs**.

Finally, the effect of KD of *NES* in Panc1 and *SOX2* in Panc89 cell variants on the response towards cytostatic drugs was determined. After 72 h transfection, reseeded cells were treated with either Gemcitabine, 5-Fluorouracil (5-FU) or Oxaliplatin for 72 h.

**Fig. 5:**
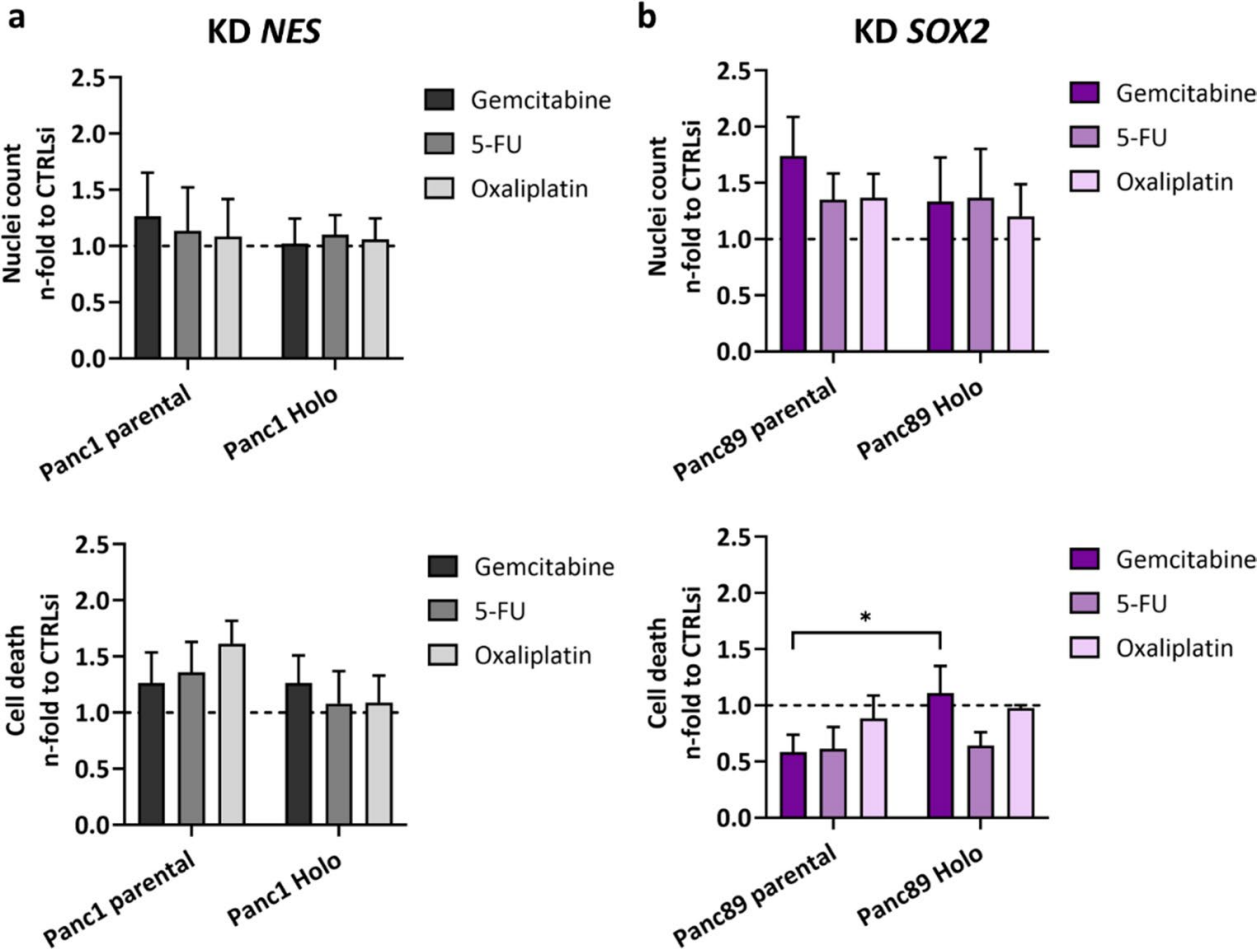
**siRNA-mediated KD of** ***NES*** **in Panc1 cell variants and** ***SOX2*** **in Panc89 cell variants marginally impacts the response to cytostatic drugs.** 5 × 10^4^ cells were subjected to siRNA-mediated KD of *NES* in Panc1 and *SOX2* Panc89 cell variants or to CTRLsi transfection for 72 h. Afterward, 1 × 10^3^ cells/well of either cell variants and conditions were re-seeded in a 96-well plate and after 24 h, the cells were treated with Gemcitabine, 5-FU or Oxaliplatin for 72 h. Then, (a) Panc1 and (b) Panc89 cell variants were stained with Hoechst 33342 (1:5000) and PI (1:50) and imaged to determine the total number of cells (Hoechst-positive, nuclei count) and the number of dead cells (Hoechst/PI-positive, cell death), respectively. Every analysis was performed with *n* = 3 independent experiments. The nuclei count and cell death values after KD of *NES* and *SOX2*, respectively, were normalized to CTRLsi conditions. All data sets were analyzed by two-way ANOVA and Tukey´s multiple comparison. Every analysis was performed with *n* = 3 independent experiments plus technical replicates and data are shown as mean with SEM. Significances are indicated by asterisk: *p* ≤ 0.033 = *, *p* ≤ 0.002 = **, *p* ≤ 0.001 = ***. (KD = knockdown; CTRLsi = control siRNA; 5-FU = 5-Fluorouracil; PI = propidium iodide; SEM = standard error of means; Holo = Holoclone)

The nuclei count analysis of both Panc1 cell variants revealed no or only marginal effects by *NES* KD on the response towards Gemcitabine, 5-FU or Oxaliplatin (Fig. 5a). The cell death analysis revealed that the number of dead cells increased up to 1.5-fold after treatment with the cytostatic drugs and *NES* KD in parental Panc1 cells, with the strongest response to Oxaliplatin treatment, while these effects were almost not observed in Panc1 Holoclone cells after *NES* KD (Fig. 5a).

In both parental Panc89 cells and Panc89 Holoclone cells the nuclei count was increased up to 1.7-fold after *SOX2* KD and treatment with the cytostatic drugs compared to CTRLsi conditions (Fig. 5b). In line with the elevated number of nuclei counts, parental Panc89 cells showed a decreased cell death rate by trend after *SOX2* KD compared to CTRLsi conditions after treatment either of the cytostatic drugs (Fig. 5b). Overall, cell death was declined in both Panc89 cell variants after *SOX2* KD and all treatments, except in Panc89 Holoclone cells after KD of *SOX2* and Gemcitabine or Oxaliplatin treatment, where almost no effect was noted (Fig. 5b).

In summary, this data indicates that neither Nestin nor SOX2 alone are decisive for the response to cytostatic drug treatment in Panc1 and Panc89 cell variants.

## Discussion

PDAC remains one of the most challenging and life-threatening malignancies [1, 2]. The high tumor cell heterogeneity and cancer cell plasticity of PDAC tumors, along with other factors such as the tumor stroma and microbiome, leads to poor prognosis of PDAC patients [36, 43, 56–65]. Cellular plasticity in tumors is characterized by the ability of cancer cells to rapidly adapt their phenotype in response to changing environments, e.g. by switching from a sessile, proliferative to a motile, invasive cell state, and vice versa [16, 66, 67]. Furthermore, these plasticity related processes are associated with the gain and loss of CSC properties [31, 33, 68, 69]. CSCs have the unique ability of self-renewal, initiation of tumoral lesions, and to resist cell death induction [32, 33, 70–73]. Altogether, these factors lead to a rapid outgrowth of PDAC tumors, a high metastatic potential as well an increased resistance against chemotherapy for PDAC patients [36, 58, 63, 74–76]. Yet, it is still poorly understood whether and how different EMT states are linked to CSC phenotypes and how this impacts malignancy associated properties.

By comprehensively characterizing PDAC cell lines and the isolated CSC and non-CSC cell variants thereof exhibiting different EMT states we could already show that different CSC-EMT phenotypes exist that are associated with distinct functional properties, e.g. self-renewal capacity, migration, invasion, therapy response and tumorigenicity [43]. While mesenchymal Panc1 cells show a Nestin-dominated CSC phenotype, epithelial Panc89 cells exhibit a SOX2-dominated CSC phenotype [43].

To gain a better understanding of the role of these distinct CSC markers on the EMT phenotype as well as functional behavior of PDAC cells, a siRNA-mediated KD of *NES* in Panc1 cells variants and *SOX2* in Panc89 cell variants, as well as a double KD of both markers in all cell lines was performed. We could demonstrate a reliable and significant reduction of gene expression and protein levels of Nestin and SOX2 in Panc1 and Panc89 cell variants, respectively. Interestingly, we observed that Panc1 cell variants showed a slight increase of *SOX2* expression under *NES* KD, and Panc89 cell variants revealed an increased *NES* expression under *SOX2* KD. These gene expression data are in line with other studies suggesting potential interactions between Nestin and SOX2 in PDAC [54, 77], but could not be reliable demonstrated on protein level. However, a previous study showed that SOX2 can bind to an enhancer region of the *NES* gene, leading to an upregulated *NES* expression [54].

Our further analysis revealed that the effect of *NES* or *SOX2* KD on the expression of EMT markers and plasticity modulators were rather minor, with an increased gene expression of the epithelial marker E-cadherin (*CDH1*) in Panc1 cell variants and in parental Panc89 cells after either KD being the strongest effect. These findings indicate a phenotypic switch towards a more epithelial-like phenotype of PDAC cells under downregulation of *NES* [50] and *SOX2*, respectively, the latter being known to suppress E-cadherin expression by binding to Snail, Slug and Twist promoters [54]. Although the double KD of *NES/SOX2* strengthened some effects on EMT markers and plasticity modulators by a further decrease in gene expression of *L1CAM*, *VIM* and *ZEB2* in Panc1 cell variants and *L1CAM*, *VIM*, *ZEB1* and *ZEB2* in Panc89 cell variants, this effect could not be validated on protein level. Overall, these results suggest a rather minor impact of either CSC marker on EMT marker expression, which is in line with the observation that neither *NES* KD nor *SOX2* KD affected cell migration and invasion of PDAC cell variants.

Functional analysis of Panc1 and Panc89 cell variants revealed decreased viable cell numbers for Panc1 cell variants under *NES* KD and KD of *NES/SOX2*, an effect being more pronounced in Panc1 Holoclone cells. This is in line with the current knowledge of Nestin being a key player in proliferation capability in different cancer entities, e.g. PDAC [78–80]. Although SOX2 is also known to maintain a cell proliferation capacity in PDAC cells [45] and a coordinated gene expression of both, *NES* and *SOX2*, is needed to promote cancer cell proliferation and survival [54, 77], *SOX2* KD as well as KD of both *NES/SOX2* had only marginal effects causing slightly decreased cell numbers in Panc89 cell variants. However, CFAs revealed that KD of *NES* in Panc1 cell variants and KD of *SOX2* in Panc89 cell variants did not alter the number of colonies formed, but led to clearly decreased formation of Holoclone colonies. As the decreased number of Holoclone colonies was not accompanied by a likewise increased number of Mero- and Paraclones, Nestin and SOX2, respectively, seem to predominantly impact the CSC phenotype and the self-renewal capabilities of all four PDAC cell variants supporting the role of these proteins in self-renewal of PDAC cells. However, the observation that KD of both *NES/SOX2* did not further intensify the effect of the respective single KD suggests that the self-renewal capacity in PDAC cells can be maintained by different mechanisms being dependent on different factors (as Nestin or SOX2). Thus, our findings also support the view that distinct CSC populations exist in PDAC being dependent on different self-renewal determining factors [30–33, 36, 37, 43].

Furthermore, either CSC marker KD only marginally impacted the migration and invasion properties of the PDAC cell variants analyzed, with *NES* KD having a rather enhancing effect by trend in parental Panc1 cells and *SOX2* KD exerting a rather inhibitory effect by trend in Panc89 cell variants.

Finally, we investigated the impact of *NES* and *SOX2* KD in Panc1 and Panc89 cell variants, respectively, on the response to clinically relevant cytostatic drugs. Nuclei count analysis revealed that *NES* KD did not alter the nuclei count of Panc1 cell variants after treatment with the chemotherapeutic drugs Gemcitabine, 5-FU or Oxaliplatin, while the number of dead cells slightly increased, especially for parental Panc1 cells. This data is in line with CSCs being more resistant to conventional therapies [76, 81]. In contrast, the number of either Panc89 cell population even increased along with decreased number of dead cells under *SOX2* KD when treated with Gemcitabine, 5-FU or Oxaliplatin. These findings contradict other studies that show that SOX2 contributes to chemoresistance in colorectal cancer cells by upregulating ATP-binding cassette transporters, which function as efflux pumps for anti-cancer drugs [53, 82]. Furthermore, it has been shown that downregulation of *SOX2* can sensitize PDAC cells to Gemcitabine, indicating a role in mediating chemoresistance in PDAC [83]. Additionally, SOX2 has been shown to promote proliferation and tumor growth in a variety of cancer entities [52, 53, 82, 84]. The fact that *SOX2* KD in the Panc89 cell variants analyzed did not elevate the response towards chemotherapeutic drugs might be explained by the concomitant upregulation of *NES* for which a role in the mediation of chemoresistance in cancer cells, e.g. hepatocellular carcinoma cells [85] or small cell lung cancer cells [86], has been already described. This data indicates a potential compensation of Nestin and SOX2 under each other’s KD. In addition, the functional effects of both CSC factors may also be context dependent and rely on other coregulatory factors expressed in the cells [47, 77, 80, 82, 83, 87–94].

Overall, our data demonstrate that Nestin and SOX2 are crucial mediators of self-renewal capabilities of mesenchymal-like and epithelial PDAC cell variants, respectively, but that further factors are required for the maintenance of other malignancy associated properties such as proliferation, migration, invasion or drug responses. Our study further supports the view that both Nestin and SOX2 essentially contributes to the aggressive nature of PDAC cells, in which their effects seem to be highly dependent on the cellular regulatory context.

## Supplementary Information

Below is the link to the electronic supplementary material.

Supplementary Material 1

## Data Availability

Data will be made available on request.
