# Supplementary material for: Nestin and SOX2 Maintain self-renewal Abilities of Different Pancreatic Cancer Stem Cell Populations
Source: Stem Cell Rev Rep. 2025 Oct 23;22(1):620–35. doi: 10.1007/s12015-025-11006-3 (PMC12795969; doi:10.1007/s12015-025-11006-3)
Supplement: Supplementary file 1 — Supplementary Material 1 [file 12015_2025_11006_MOESM1_ESM.docx]

**Supplementary Information**

**Nestin and SOX2 maintain self-renewal abilities of different pancreatic cancer stem cell populations**

Lisa-Marie Philipp^1^, Patrick Hoffmann^1^, Luisa Hattingen^1^, Amelie Modi^1^, Susanne Sebens^1#^

^1^ Institute for Experimental Cancer Research, Kiel University and University Hospital Schleswig-Holstein Campus Kiel, Kiel, Germany

# Corresponding author: [susanne.sebens@email.uni-kiel.de](mailto:susanne.sebens@email.uni-kiel.de)

**Methods**

## **Western Blot**

For the Western Blot analysis, 5×10^4^ cells/well were seeded in 12-well plates and after 24 h, cells were transfected as described above. After 72 h transfection, the transfectant containing medium was removed, cells were washed with PBS and lysed with Laemmli-buffer containing 1 mM sodium orthovanadate. After ultra sonification of the lysates, the protein concentration was determined via DC^TM^ Protein assay (Bio-Rad Laboratories, Munich, DE). Proteins (20 µg protein per sample) were separated according to their molecular weight using a discontinuous sodium dodecyl sulfate polyacrylamide gel (SDS-PAGE, 10 %) and the Mini-PROTEAN^®^ Tetra Vertical Electrophoresis Cell-System (Bio-Rad Laboratories, Munich, DE). Electrophoretic separation was performed at 120 V. Following SDS-PAGE, proteins were transferred to a polyvinylidene fluoride (PVDF) membrane according to the manufacturer's instructions (Bio-Rad Laboratories, Munich, DE) using a semi-dry method. The membrane was activated in methanol for about 1 min and briefly washed in distilled water. For the protein transfer, the filter papers were moistened with Trans-Blot^®^ Turbo™ Transfer Buffer (Bio-Rad Laboratories, Munich, DE) and a sandwich consisting of three filter papers, PVDF membrane, SDS gel, and another three filter papers was assembled and blotted in the Trans-Blot® Turbo™ (Bio-Rad Laboratories, Munich, DE). The proteins were transferred for 7 min at 2.5 A/25 V using the manufacturer's specified program for mixed molecular weights. To reduce non-specific protein binding, the membrane was blocked in 5% milk/TBS-T for 1 h at RT. The respective primary antibody (Table 3) was incubated overnight at 4°C on a roller mixer. The following day, the membrane was washed three times for 10 min with TBS-T and then incubated for 1 h at RT with the secondary antibody (Supplementary table 1). After repeated washing in TBS-T, the proteins were detected in the Fusion SL detection system (Vilber Lourmat, Eberhardzell, DE). For protein detection, the membrane was incubated for 2 min with WesternBright™ ECL (Advansta Inc., San Jose, USA). The molecular weight of the detected proteins was assigned based on the protein standard.

**Supplementary Table 1:** **Primary and secondary antibodies used for Western Blot analysis**

| Primary antibody  (clone) | Isotype | | Stock  Concentration | | | Dilution factor | | Manufacturer |
| --- | --- | --- | --- | --- | --- | --- | --- | --- |
| E‑Cadherin (32A8) | Mouse, IgG1 | | - | | | 1000 | | Cell Signaling  (Frankfurt, DE) |
| HSP 90 (F-8) | Mouse, IgG2a | | 200 mg/ml | | | 1000 | | Santa Cruz  (Biotechnology  Heidelberg, DE) |
| L1CAM (9.3) | Mouse, IgG2a | | 2.46 mg/ml | | | 1000 | | Santa Cruz  (Biotechnology  Heidelberg, DE) |
| Nestin (10C2) | Mouse, monoclonal IgG1 | | 1 mg/ml | | | 500 | | Thermo Fisher  Scientific  (Schwerte, DE) |
| SOX2 (D6D9) | Rabbit, monoclonal IgG | | - | | | 500 | | Cell Signaling  (Frankfurt, DE) |
| Vimentin (V9) | Mouse, IgG1 | | 200 µg/ml | | | 200 | | Santa Cruz  (Biotechnology  Heidelberg, DE) |
| Zeb1 (NBP1-05987) | Rabbit, polyclonal | | 200 µg/ml | | |  | | Novus Biologicals  (Wiesbaden, DE) |
| Secondary antibody | **Isotype** | **Concentration** | | | **Dilution factor** | | **Manufacturer** | |
| Anti-mouse IgG (HRP-coupled) | Horse | - | | 2000 | | | Cell Signaling  (Frankfurt, DE) | |
| Anti-rabbit IgG  (HRP-coupled) | Goat | - | | 2000 | | | Cell Signaling  (Frankfurt, DE) | |


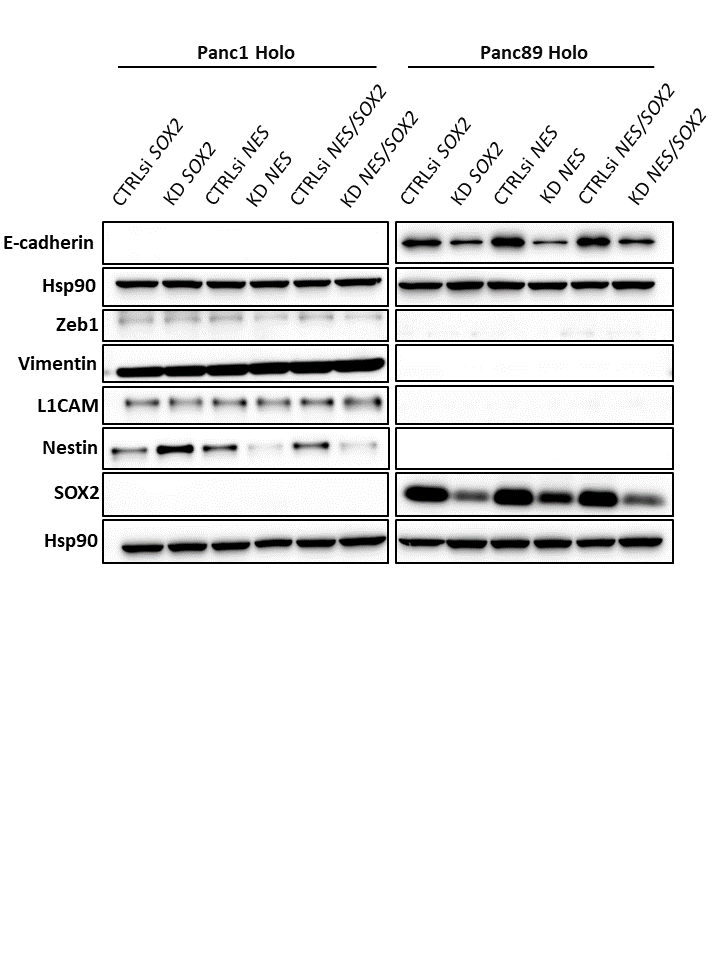


**Supplementary Figure 1: Western Blot analysis of EMT and CSC marker protein levels in Panc1 and Panc89 cell variants under KD of *NES* and *SOX2*, respectively, or double KD of *NES/SOX2*.** 5×10^4^ Panc1 or Panc89 Holoclone cells were subjected to siRNA-mediated KD of *NES, SOX2*, double KD of *NES/SOX2* or to CTRLsi transfection for 72 h. Afterward, 20 µg of protein lysate were separated according to their molecular weight via discontinuous SDS-PAGE (10 %). Proteins were transferred to a PVDF membrane via Western Blot followed by primary antibody incubation (E-Cadherin, Zeb1, Vimentin, L1CAM, Nestin, SOX2 and Hsp90, the latter used as housekeeping protein). After secondary antibody incubation, the proteins were detected and imaged. The molecular weight of the detected proteins was assigned based on the protein standard. The Western Blot image of one representative blot from n = 3 independent replicates is shown. (EMT = Epithelial-to-Mesenchymal Transition; CSC = cancer stem cell, KD = knockdown; CTRLsi = control siRNA; SDS-PAGE = sodium dodecyl sulfate-polyacrylamide gel; PVDF = polyvinylidene fluoride)
